# Can Biofilm Be Reversed Through Quorum Sensing in *Pseudomonas aeruginosa*?

**DOI:** 10.3389/fmicb.2019.01582

**Published:** 2019-07-23

**Authors:** Shaomin Yan, Guang Wu

**Affiliations:** State Key Laboratory of Non-Food Biomass and Enzyme Technology, National Engineering Research Center for Non-Food Biorefinery, Guangxi Key Laboratory of Biorefinery, Guangxi Biomass Engineering Technology Research Center, Guangxi Academy of Sciences, Nanning, China

**Keywords:** biofilm, *P. aeruginosa*, quorum sensing, transcriptome, positive feedback, negative feedback

## Abstract

*Pseudomonas aeruginosa* is a Gram-negative bacterium causing diseases in plants, animals, and humans, and its drug resistance is a major concern in medical care. Biofilms play an important role in *P. aeruginosa* drug resistance. Three factors are most important to induce biofilm: quorum sensing (QS), bis-(3′-5′)-cyclic diguanosine monophosphate (c-di-GMP), and small RNAs (sRNAs). *P. aeruginosa* has its own specific QS system (PQS) besides two common QS systems, LasI–LasR and RhlI–RhlR, in bacteria. PQS is interesting not only because there is a negative regulation from RhlR to *pqsR* but also because the null mutation in PQS leads to a reduced biofilm formation. Furthermore, *P. aeruginosa* dispersed cells have physiological features that are distinct between the planktonic cells and biofilm cells. In response to a low concentration of c-di-GMP, *P. aeruginosa* cells can disperse from the biofilms to become planktonic cells. These raise an interesting hypothesis of whether biofilm can be reversed through the QS mechanism in *P. aeruginosa*. Although a single factor is certainly not sufficient to prevent the biofilm formation, it necessarily explores such possibility. In this hypothesis, the literature is analyzed to determine the negative regulation pathways, and then the transcriptomic data are analyzed to determine whether this hypothesis is workable or not. Unexpectedly, the transcriptomic data reveal a negative regulation between *lasI* and *psqR*. Also, the individual cases from transcriptomic data demonstrate the negative regulations of PQS with *laslI, laslR*, *rhlI*, and *rhlR* under different experiments. Based on our analyses, possible strategies to reverse biofilm formation are proposed and their clinic implications are addressed.

## Introduction

*Pseudomonas aeruginosa* is a Gram-negative bacterium living in soil and water. Being an opportunistic pathogen, *P. aeruginosa* can cause the bacterial soft rot in plants (Rahme et al., 2000; Walker et al., 2004), and diseases in animals (Ferris et al., 2017; Vingopoulou et al., 2018) and humans, including eye (Willcox, 2007), burn wound (Church et al., 2006), acute and chronic pulmonary infections, where cystic fibrosis is associated with substantial morbidity and mortality (Elborn, 2016; Klockgether and Tümmler, 2017).

Therefore, *P. aeruginosa* is a major concern in medical care because of its drug resistance against the traditional antibiotic therapy (Buhl et al., 2015; Oliver et al., 2015), that is particularly problematic for immunocompromised patients and the elderly in nosocomial environments (Xia et al., 2016). *P. aeruginosa* brings about its drug resistance through hydrolyzation of antibiotics with carbapenemases or extended-spectrum β-lactamases or ApmR (Vatcheva-Dobrevska et al., 2013; Fisher and Mobashery, 2014; Hakemi Vala et al., 2014), the low permeability of outer membrane (Eren et al., 2013; Zgurskaya et al., 2015), the multidrug efflux (Poole, 2004; Aghazadeh et al., 2014), etc. Also, the biofilm is an important player in *P. aeruginosa* drug resistance (Mah et al., 2003) because the dense extracellular matrix of biofilms reduces the efficacy of detergents and antibiotics (Mah et al., 2003). Such resistance could be increased a thousand times in some cases (Stewart and Costerton, 2001).

The dispersal of cells from the biofilm colony is a crucial and unique stage for biofilms to spread and colonize new surfaces (Monroe, 2007) and for the transition of dispersed cells from the biofilm to the planktonic growth phase. Could it be possible to stop the biofilm from happening, or reserve the biofilm back to the planktonic phenotype, or eradicate the biofilm in bacteria?

Theoretically, this hypothesis could be possible for *P. aeruginosa*, because its dispersed cells have physiological features that are distinct between the planktonic and the biofilm cells (Chua et al., 2014, 2015). In response to a low concentration of c-di-GMP, *P. aeruginosa* cells can disperse from the biofilm to become the planktonic cells. The drug resistance is not stronger in the biofilm cells than in the stationary-phase planktonic cells, but is stronger than in the logarithmic-phase planktonic cells (Spoering and Lewis, 2001). Additionally, *P. aeruginosa* produces *cis*-2-decenoic acid, which is a fatty acid messenger and induces dispersion and inhibits the growth of biofilm colonies (Davies and Marques, 2009). Furthermore, nitric oxide triggers the dispersal of biofilms in *P. aeruginosa* (Barraud et al., 2006), leading to the treatment of chronic infections in cystic fibrosis (Howlin et al., 2017).

The formation of biofilm is induced and regulated by numerous genes and environmental factors (Fazli et al., 2014), of which three are most important. The first one is the quorum sensing (QS), because QS controls about 10% genes in *P. aeruginosa* (Wagner et al., 2003), including many genes that are actively involved in the biofilm development and dispersal, although they are unlikely to be involved in the attachment and the initial of biofilm growth (Davies et al., 1998). The second one is the bis-(3′-5′)-cyclic diguanosine monophosphate (c-di-GMP), because its signaling network is the most complex secondary signaling system in bacteria (Hengge, 2009) and has the responsibility to decide whether bacteria adopt either planktonic or biofilm phenotype (Jenal and Malone, 2006). The third one is the small RNAs (sRNAs) although their role in biofilm is yet to be clear (Wolska et al., 2016).

Indeed, QS has a close relationship with biofilm (Wolska et al., 2016). It controls the synthesis of rhamnolipids that maintain the channels (Stoodley et al., 1994) for distributing nutrient and oxygen and removing waste products in mushroom-shaped structures (Davey and O’Toole, 2000). The channels can help in the release of a large amount of eDNA due to the autolysis of subpopulation of bacteria (Allesen-Holm et al., 2006) at the late stage of biofilm development. Various components of the biofilm matrix, such as extracellular DNA (eDNA), exopolysaccharides (EPS) and glucan, are closely related to biofilm matrix dynamics and bacterial virulence (Rainey et al., 2019). Also, there are other virulence factors, which play an important role in the QS regulation and biofilm formation. For example, pyocyanin promotes eDNA release and facilitates the biofilm formation (Klare et al., 2016).

It is worth reviewing literature to explore whether the biofilm is theoretically reversible through QS in *P. aeruginosa*, not only because *P. aeruginosa* is a causal organism of important health ailments but also because *P. aeruginosa* is a commonly used biofilm model organism (Rasamiravaka et al., 2015). More importantly, the synthesis of rhamnolipid in *P. aeruginosa* occurs at its late-exponential and stationary phases (Guerra-Santos et al., 1986). Rhamnolipid helps bacteria to utilize long-chain fatty acids as sources of carbon (Ochsner et al., 1994a) so it plays an important role in the biofilm formation (Stoodley et al., 1994; Davey and O’Toole, 2000; Allesen-Holm et al., 2006).

Reversing of biofilms could be plausible because QS is a target in many different circumstances such as attenuate virulence (Chan et al., 2015), bacterial metabolism (Goo et al., 2015), bacterial response to antibiotics (Rasamiravaka and El Jaziri, 2016), and therapy (LaSarre and Federle, 2013). Besides, the mechanism to form biofilms in *P. aeruginosa* is definitely different from other bacteria such as *P. putida*, *P. fluorescens*, *Staphylococcus aureus*, and *Vibrio cholera* (Wolska et al., 2016).

Needless to say, the reversing of biofilms is related to multiple factors, so a single factor such as QS could have very limited effects. However, we should theoretically explore those possibilities one by one at initial stage in view of the importance of biofilms in clinical meanings.

## Positive and Negative Regulations in Qs

If we wish to reverse the biofilm through the QS, we need to find out whether the QS is reversible or not. So far overwhelmed evidence suggests that the QS is a positive feedback system, which implies that it is impossible to stop the QS once the QS is initiated. However, we have yet to know whether the ending point of QS is the biofilm formation? If this is the case, the stop of QS will either reverse the biofilm or stop the biofilm formation. To answer this issue, it is necessary to find out the negative regulation (feedback) in QS.

The QS is a cell-to-cell communication by means of production, detection, and response of chemical compounds, autoinducers, and thus the QS changes an individual or a population behavior upon the concentration of autoinducers, which are subject to the cell density (Fuqua et al., 1994).

**FIGURE 1:**
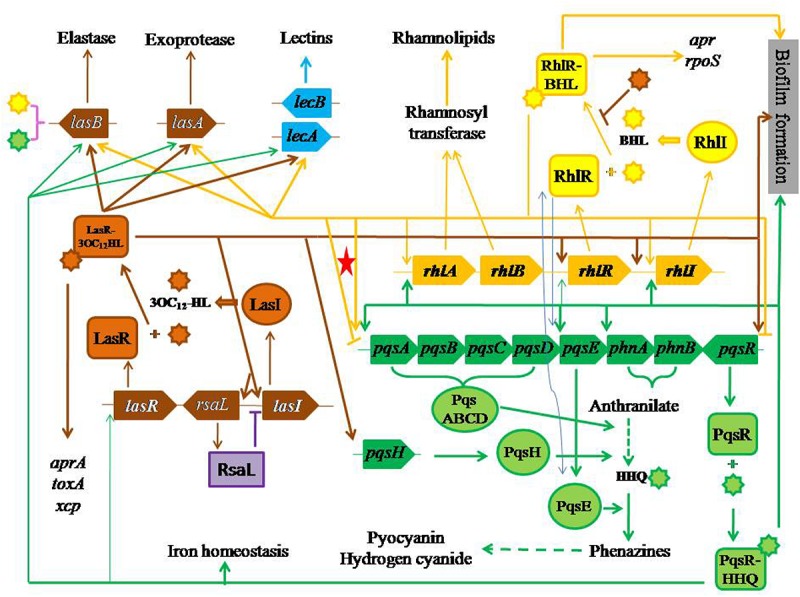
Three QS systems with their effects and regulatory pathways. The red star highlights bi-functional regulation of RhlI–RhlR system to PQS synthesis.

*Pseudomonas aeruginosa* has three QS systems. (i) LasI–LasR that is related to the synthesis and the use of *N*-(3-oxo-dodecanoyl)-L-homoserine lactone (3OC_12_-HL) (Passador et al., 1993; Pearson et al., 1994), whose concentration is ranged from 1 to 5 μM (Pearson et al., 1994, 1995) (brown color items in Figure 1). (ii) RhlI-RhlR that is related to the synthesis and the use of *N*-(butyryl)-L-homoserine lactone (BHL) (Pearson et al., 1995), whose concentration is about 10 μM (Pearson et al., 1995) (yellow color items in Figure 1). (iii) Pseudomonas quinolone signal (PQS)-based QS, PqsABCDH-PqsR that is related to the synthesis and the use of 2-heptyl-3-hydroxy-4-quinolone (HHQ) (Mashburn-Warren et al., 2008; Kulkarni and Jagannadham, 2014), whose concentration is about 6 μM (Pesci et al., 1999) (green color items in Figure 1). The first two QS systems essentially are *N*-acylated homoserine lactone (AHL)-based QS systems (Pesci et al., 1997) and exist in many bacteria.

The sophisticated QS systems in *P. aeruginosa* are described as follows. (i) LasI produces 3OC_12_-HL, which acts on LasR (Gambello and Iglewski, 1991; Pearson et al., 1994) (the upward brown arrow from *lasR* to LasR on the left side of Figure 1). (ii) LasR acts on *aprA* (Gambello et al., 1993), *lasA* (Toder et al., 1991) and *toxA* (Gambello and Iglewski, 1991; Gambello et al., 1993; Passador et al., 1993) (the downward brown arrow on the far left side in Figure 1). (iii) Both LasI and LasR act on *lasB* (Pearson et al., 1994, 1995) through 3OC_12_-HL, whose half-maximal expression needs 1.0 nM (Seed et al., 1995) (brown symbols on the left side of Figure 1). (iv) RhlI produces BHL, which acts on RhlR (Pearson et al., 1995, 1997) (the bright yellow arrow on the right side of Figure 1). (v) RhlR acts on pyocyanin synthesis (Meighen, 1991; Ochsner et al., 1994b; Brint and Ohman, 1995) (the long yellow arrow on the middle of Figure 1), *lasA* (Brint and Ohman, 1995) (the yellow arrow on the middle left of Figure 1), and *rpoS* (Latifi et al., 1996) (the yellow arrow on the upper right corner of Figure 1). (vi) Both RhlI and RhlR act on *lasB* through BHL (Brint and Ohman, 1995) (the yellow arrow on the upper right part of Figure 1), and *rhlABR* (Ochsner and Reiser, 1995) (the small yellow arrow on the middle of Figure 1), where *rhlAB* encodes rhamnosyltransferase (Ochsner et al., 1994a) (two yellow arrows on the upper middle part of Figure 1) together with *rhlR* positively regulate rhamnolipid synthesis (Ochsner et al., 1994b) (the yellow arrow on the middle upper part of Figure 1). (vii) LasR and RhlR positively regulate the synthesis of hydrogen cyanide (Pessi and Haas, 2000) (the downward yellow arrow on the lower middle part of Figure 1).

Still, Figure 1 displays the effects of PQS-based QS on their targets. (i) PqsABCDH produces HHQ requiring *phnA* and *phnB* through anthranilate (Gallagher et al., 2002) (the green curly lines on the middle right part of Figure 1), then HHQ acts on PqsR (Cao et al., 2001), regulating the production of elastase, PA-IL lectin, pyocyanin and rhamnolipid (Pesci et al., 1999; McKnight et al., 2000; Gallagher et al., 2002; Lee and Zhang, 2015) (the green lines from the lower right corner in Figure 1). (ii) PqsE positively acts on biosynthesis of various virulent factors, which is independent of HHQ or any compounds produced related to the function of *pqsABCDE* operon although the expression of *pqsE* and PqsE are controlled by HHQ and PqsR (Farrow, et al., 2008) (dashed green line on the lower right part of Figure 1). (iii) PqsR–HHQ is involved in iron homeostasis (Bredenbruch et al., 2006; Oglesby et al., 2008) (the lowest green line in Figure 1).

A positive feedback can be found in each of three QS systems. (i) The first positive feedback goes from LasR–3OC_12_-HL to LasI through *lasI*, whose half-maximal expression needs 0.1 nM 3OC_12_-HL (Seed et al., 1995) (light gray ellipse on the left part of Figure 1). (ii) The second positive feedback goes from RhlR–HL to RhlI through *rhlI* (Ochsner and Reiser, 1995) (light gray ellipse on the upper right part of Figure 1). (iii) The third positive feedback goes from PqsR–HHQ to *pqsABCDE* and *phnAB* operons (Cao et al., 2001; Gallagher et al., 2002; Wade et al., 2005) (light gray ellipse on the middle right part of Figure 1).

The relationship among three QS systems in *P. aeruginosa* is positive in the following regulations. (i) LasR positively regulates HHQ through the complex LasR–3OC_12_-HL on *pqsH* (Pesci et al., 1999; Schertzer et al., 2009) (brown arrow on the right middle part of Figure 1). (ii) RhlR positively regulates HHQ through *PqsE* (Pesci et al., 1999) (two arrow-blue lines on the middle right part of Figure 1). (iii) LasR positively regulates *rhlR* through the complex LasR–3OC_12_-HL (Latifi et al., 1996; Pesci et al., 1997) and *rhlI* (Latifi et al., 1996) (the brown horizontal line with two arrows in Figure 1). (iv) HHQ strongly acts on *rhlI* with BHL (two arrow-blue lines in middle right part of Figure 1) but weakly acts on *lasR* and *rhlR* (McKnight et al., 2000). (v) RhlR positively regulates PqsE, whose overexpression leads to a high rhamnolipid production (Farrow, et al., 2008) (the yellow arrow on the right middle part of Figure 1). (vi) PqsE changes the function of RhlR rather than that of BHL (Farrow, et al., 2008) (two arrow-blue lines on the middle right part of Figure 1). (vii) LasR/3OC_12_-HL controls *pqsR* (Camilli and Bassler, 2006) (the end arrow of brown horizontal line in Figure 1).

In fact, there is a negative regulation among QS systems, namely, RhlR negatively regulates *pqsR* in *P. aeruginosa* (Pesci et al., 1997; Wade et al., 2005), or RhlR and BHL together negatively affect the production of HHQ and other quinolones through *pqsR* to *pqsABCDE* operon transcription (McGrath et al., 2004; Jensen et al., 2006; Xiao et al., 2006b) (the yellow arrow from RhlR–BHL to the yellow horizontal line to the right end with downward dash end in Figure 1). On the other hand, a negatively regulatory pathway is not so sure (the yellow end line highlighted with a red star on the middle of Figure 1).

## Can This Negative Regulation Work?

As PQS-based QS is so particularly relevant to *Pseudomonas*, its significance should not be ignored. This is because the null mutation in PQS leads to a reduced biofilm formation and decreased the productions of pyocyanin, elastase, PA-IL lectin and rhamnolipids (Rahme et al., 1997, 2000; Cao et al., 2001; Diggle et al., 2003). Indeed, PQS directly or indirectly controls 92 or 143 genes as shown in two transcriptomic analyses (Deziel et al., 2005, Bredenbruch et al., 2006). By contrast, the other two QS systems together influence the expression in 200-plus genes (Whiteley et al., 1999).

For PQS, it does not reach its maximal production until the late stationary phase of growth (McKnight et al., 2000). This implies that HHQ is not involved in sensing the cell density, so the observation that the QS response is not reversed for small decreases in population density in *P. aeruginosa* (Williams and Camara, 2009) is not the failure of PQS. An important time interval appears between QS systems, i.e., BHL is produced during the log phase of growth but HHQ is produced during late time in the stationary phase of growth (McKnight et al., 2000), so the positive regulation of HHQ on *rhlI* is more likely to be related to the second round of RhlI cycle. If HHQ would not function at this time interval, perhaps the QS would stop.

Another promising point is that the phenazine production requires HHQ in *P. aeruginosa* (McKnight et al., 2000; Mavrodi et al., 2001). In fact, phenazines may have a significant ecological impact on the biofilm formation in *P. aeruginosa* as well as other bacteria persisting in biofilms mixed with *P. aeruginosa*. Through affecting H_2_O_2_ generation, phenazines bring about the lysis of competing bacterial cells in mixed biofilms and the subsequent eDNA release (Das and Manefield, 2013).

Perhaps, one of the best ways to explore the possibility of whether the QS is reversible through PQS in *P. aeruginosa* is to analyze the transcriptomic data in order to find some common patterns. Accordingly, we analyzed the transcriptomic data on Affymetrix *P. aeruginosa* array with 5549 *P. aeruginosa* genes, platform GPL84, from Gene Expression Omnibus (GEO) (Edgar et al., 2002; Barrett et al., 2013), including all the data in 104 publications (Supplementary Information) with 274 datasets. Each dataset represents the response to a specifically experimental condition. With these all available transcriptomic data, we wish to determine if PQS could be depressed under different experimental conditions.

Table 1 shows correlation coefficients between any two genes of three QS systems. The rationale is that there are up-regulations and down-regulations in transcriptomic data. The correlation between two genes, which are both up-regulated or both down-regulated, would suggest a positive regulation with a positive correlation coefficient. By contrast, the correlation between two genes, which are regulated oppositely, would suggest a negative regulation with a negative correlation coefficient.

**TABLE 1:** Correlation coefficient between any two genes of three QS systems using transcriptomic data.

**Gene**		***lasI***	***lasR***	***rhlI***	***rhlR***	***rhl***	***pqsA***	***pqsB***	***pqsC***	***pqsD***	***pqsE***	***pqsH***	***pqsR/mvfR***
		PA1432	PA1430	PA3476	PA3477	PA3861	PA0996	PA0997	PA0998	PA0999	PA1000	PA2587	PA1003
*lasI*	PA1432	1	0.62	0.37	0.37	0.16	0.06	0.04	−0.02	−0.01	0.01	0.17	−0.38
*lasR*	PA1430		1	0.51	0.33	0.52	0.15	0.12	0.11	0.13	0.10	0.14	−0.03
*rhlI*	PA3476			1	0.89	0.29	0.73	0.76	0.58	0.67	0.58	0.20	0.17
*rhlR*	PA3477				1	0.13	0.84	0.88	0.66	0.76	0.68	0.05	−0.04
*rhl*	PA3861					1	0.15	0.14	0.19	0.22	0.14	0.16	0.24
*pqsA*	PA0996						1	0.97	0.85	0.95	0.82	−0.05	0.25
*pqsB*	PA0997							1	0.89	0.96	0.84	−0.03	0.24
*pqsC*	PA0998								1	0.90	0.87	−0.03	0.28
*pqsD*	PA0999									1	0.81	0.00	0.30
*pqsE*	PA1000										1	−0.07	0.23
*pqsH*	PA2587											1	0.27
*pqsR/mvfR*	PA1003												1

Based upon the correlations within a single QS system in Table 1, the correlations between *lasI* and *lasR*, and between *rhlI* and *rhlR* confirm their auto-induction relationships (Gambello and Iglewski, 1991; Pearson et al., 1994) within each QS system. No negative correlation is found between the QS genes in the same QS system. As *pqsR* is named *mvfR* in gene bank, the auto-induction relationship with the rest of PQS genes are not very evident as the paired correlations between *pdsA*, *pqsB*, *pqsC*, *pqsD*, *pqsE*, but all paired correlations suggest a positive regulation within PQS system (Gallagher et al., 2002). Based upon the correlations between two QS systems in Table 1, the results conform there is a positive regulation between *lasI–lasR* and *rhlI–rhlR*, and between *rhlI-rhlR* and *pqsABCDE*. However, an undocumented negative regulation is revealed between *lasI* and *pqsR/mvfR* using these transcriptomic data. Could it be a potential pathway to reverse the biofilm formation?

**FIGURE 2:**
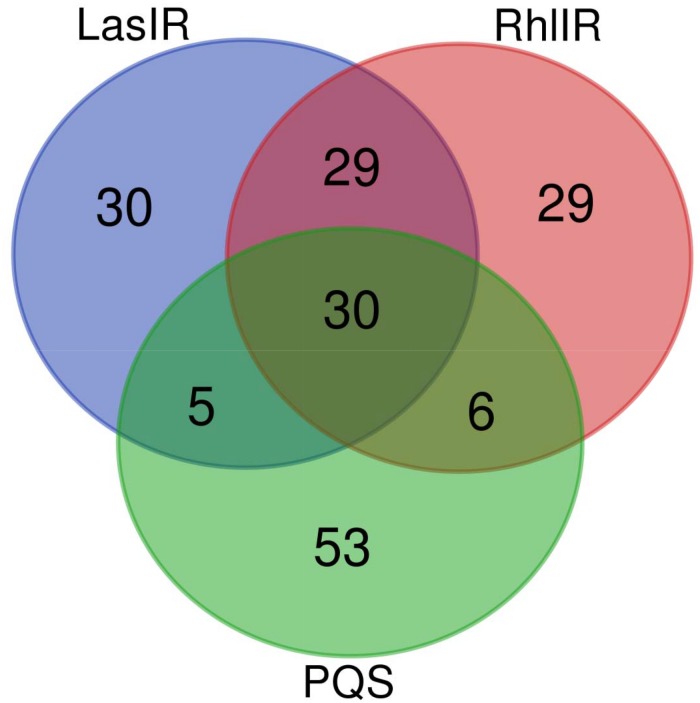
Number of transcriptomic studies affecting QS in *P. aeruginosa* PAO1. The figure was made using online server at http://bioinformatics.psb. ugent.be/webtools/Venn/.

Furthermore, the responses of QS systems are analyzed under different transcriptomic experiments, and classified as down-regulation, down-regulation/no response, no response, no response/up-regulation, up-regulation and mixed responses. Figure 2 shows such analysis according to 94 transcriptomic experiments. No response on three QS systems was found in 30 transcriptomic experiments (the intersection of three circles in Figure 2). Both LasI-LasR and RhlI-RhlR have the same response in 29 transcriptomic experiments (the intersection of two upper circles in Figure 2), suggesting a good cooperation between them. By the contrast, only five and six transcriptomic experiments show the same response for PQS with LasI-LasR and RhlI-RhlR (the intersection of two upper and lower circles in Figure 2), respectively. The same response in both LasI-LasR and PQS systems includes no response in GSE24784, GSE26142, GSE35248, and GSE39044, and no response/up-regulation in GSE22684, indicating few positive impact of LasI-LasR on PQS. The same response in both RhlI-RhlR and PQS systems includes: down-regulation in GSE9255; down-regulation/no response in GSE5887; no response in GSE17179 and GSE61925; and no response/up-regulation in GSE65882 and GSE7402. Thus, the results from Venn diagram indicate that RhlI-RhlR has weak impacts on PQS. Figure 2 demonstrates the responses of 30, 29, and 53 transcriptomic experiments solely in LasI-LasR, RhlI-RhlR, and PQS, respectively, of which their response ranges from down-regulation to mixed response.

Finally, the negative regulation between different QS systems is found in four transcriptomic experiments (GSE4152, GSE8408, GSE6122, and GSE17296). In the study on Australian clonal strain (AES-1) in patients with cystic fibrosis in GSE6122 (Manos et al., 2009), *lasI*, *rhlI*, and *rhlR* were down-regulated while *pqsA*, *pqsB*, *pqsC*, *pqsD*, and *pqsE* were up-regulated. This highlights the PQS remarkable effect on the biofilm formation and enhanced infectivity. Another three transcriptomic experiments show that *pqsA*, *pqsB*, *pqsC*, *pqsD*, and *pqsE* were down-regulated whereas *rhlI* and *rhlR* were up-regulated (Teitzel et al., 2006; Tralau et al., 2007; Kawakami et al., 2010), of which *lasI* was up-regulated in copper-stressed (Teitzel et al., 2006), and both *lasI* and *lasR* were up-regulated in sulfate limitation (Tralau et al., 2007). Therefore, RhlI-RhlR does have a negative regulation on PQS (McGrath et al., 2004; Wade et al., 2005; Xiao et al., 2006a).

## Conclusion

In this hypothesis, we apply the transcriptomic data to verify the hypothesis of whether the biofilm can be reversed in *P. aeruginosa* through QS because there are negative regulations between PQS and RhlI-RhlR. Interestingly, the transcriptomic data from 104 publications reveal a negative regulation between *lasI* and *psqR*, rendering a support to the hypothesis. Individual cases from transcriptomic data under different experiments demonstrate the negative regulations of PQS with *laslI, laslR*, *rhlI*, and *rhlR*.

In general, the relationships among different QS systems reveal positive regulations, which act together to promote the biofilm formation. However, the present analyses from literature and transcriptomic data provide the evidence that both LaslI-LaslR and RhlI-RhlR systems have negatively regulatory effects on PQS system. This is very important because these negative regulations lay the foundation for the biofilm reversion through QS. Although the exact pathways are still not fully discovered, the *N*-acylated homoserine lactone (AHL)-based QS systems can influence PQS-based QS system by inhibiting the expression of *pqsABCDE* operon and *pqsR*, resulting in the reduction of HHQ and PqsR synthesis. Consequently, the low concentration of PQS related products cannot maintain the biofilm, leading to its reversion. On the other hand, the down-regulated PQS-based QS system cannot perform well their function of positive regulations on LaslI-LaslR and RhlI-RhlR systems, which will further affect the biofilm formation, especially in the second round of RhlI cycle. Surely, there are other factors that play roles in the formation of drug-resistant multicellular biofilms, such as c-di-GMP. As mentioned in section “Introduction,” this signal can govern bacterial cells to adopt either planktonic phenotype or biofilm formation (Hengge, 2009). Recent study demonstrates that high levels of cAMP lead to the decrease of c-di-GMP content, which inhibits the biofilm formation in *P. aeruginosa* (Almblad et al., 2019).

In clinic therapeutics for infectious diseases, antibiotic resistance has been spreading widely and rapidly, which becomes a major challenge for modern medicine. The strategy of interfering the biofilm formation is effective through bacterial cell-to-cell communication, especially with QS system (Soheili et al., 2019). More recently, QS inhibitors are drawing great attention in blocking the pathogenicity from *P. aeruginosa* (Calvert et al., 2018; Schütz and Empting, 2018). The transcriptional regulator PqsR becomes an attractive object and is considered to be one of the most appropriate targets. Currently, QS regulation mechanism in *P. aeruginosa* is mainly related to positive and negative regulation between QS systems. Clearly, exploration of regulation beyond QS should get attention in future.

## Data Availability

All datasets generated or analyzed for this study are included in the manuscript and the Supplementary Files.

## Author Contributions

GW designed the work. Both authors prepared and approved the manuscript.

## Conflict of Interest Statement

The authors declare that the research was conducted in the absence of any commercial or financial relationships that could be construed as a potential conflict of interest.
