# Supplementary material for: Can Biofilm Be Reversed Through Quorum Sensing in Pseudomonas aeruginosa?
Source: Front Microbiol. 2019 Jul 23;10:1582. doi: 10.3389/fmicb.2019.01582 (PMC6664025; doi:10.3389/fmicb.2019.01582)
Supplement: Supplementary file 1 [file Data_Sheet_1.PDF]

104 Published Studies, whose transcriptomic data are used.

1. Nalca Y, Jänsch L, Bredenbruch F, Geffers R, Buer J, Häussler S. Quorum-sensing antagonistic activities of azithromycin in *Pseudomonas aeruginosa* PAO1: a global approach. Antimicrob Agents Chemother. 2006; 50(5):1680-8.
2. Zheng P, Sun J, Geffers R, Zeng AP. Functional characterization of the gene PA2384 in large-scale gene regulation in response to iron starvation in *Pseudomonas aeruginosa*. J Biotechnol. 2007; 132(4):342-52.
3. Chang W, Small DA, Toghrol F, Bentley WE. Microarray analysis of *Pseudomonas aeruginosa* reveals induction of pyocin genes in response to hydrogen peroxide. BMC Genomics. 2005; 6:115.
4. Bredenbruch F, Geffers R, Nimtz M, Buer J, Häussler S. The *Pseudomonas aeruginosa* quinolone signal (PQS) has an iron-chelating activity. Environ Microbiol. 2006; 8(8):1318-29.
5. Lequette Y, Lee JH, Ledgham F, Lazdunski A, Greenberg EP. A distinct QscR regulon in the *Pseudomonas aeruginosa* quorum-sensing circuit. J Bacteriol. 2006; 188(9):3365-70.
6. Teitzel GM, Geddie A, De Long SK, Kirisits MJ, Whiteley M, Parsek MR. Survival and growth in the presence of elevated copper: transcriptional profiling of copper-stressed *Pseudomonas aeruginosa*. J Bacteriol. 2006; 188(20):7242-56.
7. Chugani S, Greenberg EP. The influence of human respiratory epithelia on *Pseudomonas aeruginosa* gene expression. Microb Pathog. 2007; 42(1):29-35.
8. Cirz RT, O'Neill BM, Hammond JA, Head SR, Romesberg FE. Defining the *Pseudomonas aeruginosa* SOS response and its role in the global response to the antibiotic ciprofloxacin. J Bacteriol. 2006; 188(20):7101-10.
9. Muller JF, Stevens AM, Craig J, Love NG. Transcriptome analysis reveals that multidrug efflux genes are upregulated to protect *Pseudomonas aeruginosa* from pentachlorophenol stress. Appl Environ Microbiol. 2007; 73(14):4550-8.
10. Attila C, Ueda A, Cirillo SL, Cirillo JD, Chen W, Wood TK. *Pseudomonas aeruginosa* PAO1 virulence factors and poplar tree response in the rhizosphere. Microb Biotechnol. 2008; 1(1):17-29.
11. Manos J, Arthur J, Rose B, Bell S, Tingpej P, Hu H, Webb J, Kjelleberg S, Gorrell MD, Bye P, Harbour C. Gene expression characteristics of a cystic fibrosis epidemic strain of *Pseudomonas aeruginosa* during biofilm and planktonic growth. FEMS Microbiol Lett. 2009 Mar;292(1):107-14.
12. Alvarez-Ortega C, Harwood CS. Responses of *Pseudomonas aeruginosa* to low oxygen indicate that growth in the cystic fibrosis lung is by aerobic respiration. Mol Microbiol. 2007; 65(1):153-65.
13. Alhede M, Bjarnsholt T, Jensen PØ, Phipps RK, Moser C, Christophersen L, Christensen LD, van Gennip M, Parsek M, Høiby N, Rasmussen TB, Givskov M. *Pseudomonas aeruginosa* recognizes and responds aggressively to the presence of polymorphonuclear leukocytes. Microbiology. 2009 Nov;155(Pt 11):3500-8.
14. Small DA, Chang W, Toghrol F, Bentley WE. Comparative global transcription analysis of sodium hypochlorite, peracetic acid, and hydrogen peroxide on *Pseudomonas aeruginosa*. Appl Microbiol Biotechnol. 2007; 76(5):1093-105.
15. Son MS, Matthews WJ Jr, Kang Y, Nguyen DT, Hoang TT. In vivo evidence of *Pseudomonas*

aeruginosa nutrient acquisition and pathogenesis in the lungs of cystic fibrosis patients. Infect Immun. 2007 Nov;75(11):5313-24.

16. Kang Y, Nguyen DT, Son MS, Hoang TT. The *Pseudomonas aeruginosa* PsrA responds to long-chain fatty acid signals to regulate the fadBA5 beta-oxidation operon. Microbiology. 2008; 154(Pt 6):1584-98.
17. Tralau T, Vuilleumier S, Thibault C, Campbell BJ, Hart CA, Kertesz MA. Transcriptomic analysis of the sulfate starvation response of *Pseudomonas aeruginosa*. J Bacteriol. 2007; 189(19):6743-50.
18. Skindersoe ME, Alhede M, Phipps R, Yang L, Jensen PO, Rasmussen TB, Bjarnsholt T, Tolker-Nielsen T, Høiby N, Givskov M. Effects of antibiotics on quorum sensing in *Pseudomonas aeruginosa*. Antimicrob Agents Chemother. 2008; 52(10):3648-63.
19. Attila C, Ueda A, Wood TK. PA2663 (PpyR) increases biofilm formation in *Pseudomonas aeruginosa* PAO1 through the psl operon and stimulates virulence and quorum-sensing phenotypes. Appl Microbiol Biotechnol. 2008; 78(2):293-307.
20. Ueda A, Attila C, Whiteley M, Wood TK. Uracil influences quorum sensing and biofilm formation in *Pseudomonas aeruginosa* and fluorouracil is an antagonist. Microb Biotechnol. 2009; 2(1):62-74.
21. Rao J, DiGiandomenico A, Unger J, Bao Y, Polanowska-Grabowska RK, Goldberg JB. A novel oxidized low-density lipoprotein-binding protein from *Pseudomonas aeruginosa*. Microbiology. 2008; 154(Pt 2):654-65.
22. Llamas MA, Mooij MJ, Sparrius M, Vandenbroucke-Grauls CM, Ratledge C, Bitter W. Characterization of five novel *Pseudomonas aeruginosa* cell-surface signalling systems. Mol Microbiol. 2008; 67(2):458-72.
23. Chou HT, Kwon DH, Hegazy M, Lu CD. Transcriptome analysis of agmatine and putrescine catabolism in *Pseudomonas aeruginosa* PAO1. J Bacteriol. 2008 Mar;190(6):1966-75.
24. Anderson GG, Moreau-Marquis S, Stanton BA, O'Toole GA. In vitro analysis of tobramycin-treated *Pseudomonas aeruginosa* biofilms on cystic fibrosis-derived airway epithelial cells. Infect Immun. 2008; 76(4):1423-33.
25. Lee J, Attila C, Cirillo SL, Cirillo JD, Wood TK. Indole and 7-hydroxyindole diminish *Pseudomonas aeruginosa* virulence. Microb Biotechnol. 2009; 2(1):75-90.
26. Manos J, Arthur J, Rose B, Tingpej P, Fung C, Curtis M, Webb JS, Hu H, Kjelleberg S, Gorrell MD, Bye P, Harbour C. Transcriptome analyses and biofilm-forming characteristics of a clonal *Pseudomonas aeruginosa* from the cystic fibrosis lung. J Med Microbiol. 2008; 57(Pt 12):1454-65.
27. Hogardt M, Hoboth C, Schmoldt S, Henke C, Bader L, Heesemann J. Stage-specific adaptation of hypermutable *Pseudomonas aeruginosa* isolates during chronic pulmonary infection in patients with cystic fibrosis. J Infect Dis. 2007; 195(1):70-80.
28. Nde CW, Jang HJ, Toghrol F, Bentley WE. Toxicogenomic response of *Pseudomonas aeruginosa* to ortho-phenylphenol. BMC Genomics. 2008 Oct 10;9:473.
29. Weir TL, Stull VJ, Badri D, Trunck LA, Schweizer HP, Vivanco J. Global gene expression profiles suggest an important role for nutrient acquisition in early pathogenesis in a plant model of *Pseudomonas aeruginosa* infection. Appl Environ Microbiol. 2008; 74(18):5784-91.
30. Mikkelsen H, Bond NJ, Skindersoe ME, Givskov M, Lilley KS, Welch M. Biofilms and type III secretion are not mutually exclusive in *Pseudomonas aeruginosa*. Microbiology. 2009;

155(Pt 3):687-98.
31. Kai T, Tateda K, Kimura S, Ishii Y, Ito H, Yoshida H, Kimura T, Yamaguchi K. A low concentration of azithromycin inhibits the mRNA expression of N-acyl homoserine lactone synthesis enzymes, upstream of lasI or rhlI, in *Pseudomonas aeruginosa*. Pulm Pharmacol Ther. 2009; 22(6):483-6.
32. Cummins J, Reen FJ, Baysse C, Mooij MJ, O'Gara F. Subinhibitory concentrations of the cationic antimicrobial peptide colistin induce the pseudomonas quinolone signal in *Pseudomonas aeruginosa*. Microbiology. 2009; 155(Pt 9):2826-37.
33. Hegde M, Wood TK, Jayaraman A. The neuroendocrine hormone norepinephrine increases *Pseudomonas aeruginosa* PA14 virulence through the las quorum-sensing pathway. Appl Microbiol Biotechnol. 2009; 84(4):763-76.
34. Ueda A, Wood TK. Connecting quorum sensing, c-di-GMP, pel polysaccharide, and biofilm formation in *Pseudomonas aeruginosa* through tyrosine phosphatase TpbA (PA3885). PLoS Pathog. 2009; 5(6):e1000483.
35. Nde CW, Jang HJ, Toghrol F, Bentley WE. Global transcriptomic response of *Pseudomonas aeruginosa* to chlorhexidine diacetate. Environ Sci Technol. 2009 Nov 1;43(21):8406-15.
36. Llamas MA, van der Sar A, Chu BC, Sparrius M, Vogel HJ, Bitter W. A Novel extracytoplasmic function (ECF) sigma factor regulates virulence in *Pseudomonas aeruginosa*. PLoS Pathog. 2009; 5(9):e1000572.
37. Crabbé A, Pycke B, Van Houdt R, Monsieurs P, Nickerson C, Leys N, Cornelis P. Response of *Pseudomonas aeruginosa* PAO1 to low shear modelled microgravity involves AlgU regulation. Environ Microbiol. 2010; 12(6):1545-64.
38. Déziel E, Gopalan S, Tampakaki AP, Lépine F, Padfield KE, Saucier M, Xiao G, Rahme LG. The contribution of MvfR to *Pseudomonas aeruginosa* pathogenesis and quorum sensing circuitry regulation: multiple quorum sensing-regulated genes are modulated without affecting lasRI, rhlRI or the production of N-acyl-L-homoserine lactones. Mol Microbiol. 2005; 55(4):998-1014.
39. Lesic B, Lépine F, Déziel E, Zhang J, Zhang Q, Padfield K, Castonguay MH, Milot S, Stachel S, Tzika AA, Tompkins RG, Rahme LG. Inhibitors of pathogen intercellular signals as selective anti-infective compounds. PLoS Pathog. 2007;3(9):1229-39.
40. Lesic B, Starkey M, He J, Hazan R, Rahme LG. Quorum sensing differentially regulates *Pseudomonas aeruginosa* type VI secretion locus I and homologous loci II and III, which are required for pathogenesis. Microbiology. 2009; 155(Pt 9):2845-55.
41. Trunk K, Benkert B, Quäck N, Münch R, Scheer M, Garbe J, Jänsch L, Trost M, Wehland J, Buer J, Jahn M, Schobert M, Jahn D. Anaerobic adaptation in *Pseudomonas aeruginosa*: definition of the Anr and Dnr regulons. Environ Microbiol. 2010; 12(6):1719-33.
42. Kawakami T, Kuroki M, Ishii M, Igarashi Y, Arai H. Differential expression of multiple terminal oxidases for aerobic respiration in *Pseudomonas aeruginosa*. Environ Microbiol. 2010; 12(6):1399-412.
43. Fung C, Naughton S, Turnbull L, Tingpej P, Rose B, Arthur J, Hu H, Harmer C, Harbour C, Hassett DJ, Whitchurch CB, Manos J. Gene expression of *Pseudomonas aeruginosa* in a mucin-containing synthetic growth medium mimicking cystic fibrosis lung sputum. J Med Microbiol. 2010; 59(Pt 9):1089-100.
44. Lee JH, Cho MH, Lee J. 3-indolylacetonitrile decreases Escherichia coli O157:H7 biofilm

formation and *Pseudomonas aeruginosa* virulence. Environ Microbiol. 2011; 13(1):62-73.
45. Huse HK, Kwon T, Zlosnik JE, Speert DP, Marcotte EM, Whiteley M. Parallel evolution in *Pseudomonas aeruginosa* over 39,000 generations in vivo. MBio. 2010; 1(4). pii: e00199-10.
46. Folsom JP, Richards L, Pitts B, Roe F, Ehrlich GD, Parker A, Mazurie A, Stewart PS. Physiology of *Pseudomonas aeruginosa* in biofilms as revealed by transcriptome analysis. BMC Microbiol. 2010 Nov 17;10:294.
47. Koh AY, Mikkelsen PJ, Smith RS, Coggshall KT, Kamei A, Givskov M, Lory S, Pier GB. Utility of in vivo transcription profiling for identifying *Pseudomonas aeruginosa* genes needed for gastrointestinal colonization and dissemination. PLoS One. 2010; 5(12):e15131.
48. Crabbé A, Schurr MJ, Monsieurs P, Morici L, Schurr J, Wilson JW, Ott CM, Tsaprailis G, Pierson DL, Stefanyshyn-Piper H, Nickerson CA. Transcriptional and proteomic responses of *Pseudomonas aeruginosa* PAO1 to spaceflight conditions involve Hfq regulation and reveal a role for oxygen. Appl Environ Microbiol. 2011; 77(4):1221-30.
49. Kwan JC, Meickle T, Ladwa D, Teplitski M, Paul V, Luesch H. Lyngbyoic acid, a "tagged" fatty acid from a marine cyanobacterium, disrupts quorum sensing in *Pseudomonas aeruginosa*. Mol Biosyst. 2011; 7(4):1205-16.
50. Bielecki P, Puchałka J, Wos-Oxley ML, Loessner H, Glik J, Kawecki M, Nowak M, Tümmler B, Weiss S, dos Santos VA. In-vivo expression profiling of *Pseudomonas aeruginosa* infections reveals niche-specific and strain-independent transcriptional programs. PLoS One. 2011;6(9):e24235.
51. Reen FJ, Haynes JM, Mooij MJ, O'Gara F. A non-classical LysR-type transcriptional regulator PA2206 is required for an effective oxidative stress response in *Pseudomonas aeruginosa*. PLoS One. 2013;8(1):e54479.
52. Yamamoto K, Arai H, Ishii M, Igarashi Y. Trade-off between oxygen and iron acquisition in bacterial cells at the air-liquid interface. FEMS Microbiol Ecol. 2011; 77(1):83-94.
53. Warren AE, Boulianne-Larsen CM, Chandler CB, Chiotti K, Kroll E, Miller SR, Taddei F, Sermet-Gaudelus I, Ferroni A, McInnerney K, Franklin MJ, Rosenzweig F. Genotypic and phenotypic variation in *Pseudomonas aeruginosa* reveals signatures of secondary infection and mutator activity in certain cystic fibrosis patients with chronic lung infections. Infect Immun. 2011; 79(12):4802-18
54. Sonnleitner E, Gonzalez N, Sorger-Domenigg T, Heeb S, Richter AS, Backofen R, Williams P, Hüttenhofer A, Haas D, Bläsi U. The small RNA PhrS stimulates synthesis of the *Pseudomonas aeruginosa* quinolone signal. Mol Microbiol. 2011; 80(4):868-85
55. Bielecki P, Komor U, Bielecka A, Müsken M, Puchałka J, Pletz MW, Ballmann M, Martins dos Santos VA, Weiss S, Häussler S. Ex vivo transcriptional profiling reveals a common set of genes important for the adaptation of *Pseudomonas aeruginosa* to chronically infected host sites. Environ Microbiol. 2013; 15(2):570-87
56. Amini S, Hottes AK, Smith LE, Tavazoie S. Fitness landscape of antibiotic tolerance in *Pseudomonas aeruginosa* biofilms. PLoS Pathog. 2011; 7(10):e1002298.
57. Chu W, Zere TR, Weber MM, Wood TK, Whiteley M, Hidalgo-Romano B, Valenzuela E Jr, McLean RJ. Indole production promotes Escherichia coli mixed-culture growth with *Pseudomonas aeruginosa* by inhibiting quorum signaling.
58. Bobadilla Fazzini RA, Skindersoe ME, Bielecki P, Puchałka J, Givskov M, Martins sos Santos VA. Protoanemonin: a natural quorum sensing inhibitor that selectively activates iron starvation

response. Environ Microbiol. 2013; 15(1):111-20.

59. Kim YG, Lee JH, Kim CJ, Lee JC, Ju YJ, Cho MH, Lee J. Antibiofilm activity of Streptomyces sp. BFI 230 and Kribbella sp. BFI 1562 against *Pseudomonas aeruginosa*. Appl Microbiol Biotechnol. 2012; 96(6):1607-17.
60. Petrova OE, Schurr JR, Schurr MJ, Sauer K. The novel *Pseudomonas aeruginosa* two-component regulator BfmR controls bacteriophage-mediated lysis and DNA release during biofilm development through PhdA. Mol Microbiol. 2011; 81(3):767-83.
61. Sheng L, Pu M, Hegde M, Zhang Y, Jayaraman A, Wood TK. Interkingdom adenosine signal reduces *Pseudomonas aeruginosa* pathogenicity. Microb Biotechnol. 2012; 5(4):560-72.
62. Romanowski K, Zaborin A, Fernandez H, Poroyko V, Valuckaite V, Gerdes S, Liu DC, Zaborina OY, Alverdy JC. Prevention of siderophore- mediated gut-derived sepsis due to P. aeruginosa can be achieved without iron provision by maintaining local phosphate abundance: role of pH. BMC Microbiol. 2011; 11:212.
63. Zaborin A, Gerdes S, Holbrook C, Liu DC, Zaborina OY, Alverdy JC. *Pseudomonas aeruginosa* overrides the virulence inducing effect of opioids when it senses an abundance of phosphate. PLoS One. 2012;7(4):e34883.
64. Zaborin A, Romanowski K, Gerdes S, Holbrook C, Lepine F, Long J, Poroyko V, Diggle SP, Wilke A, Righetti K, Morozova I, Babrowski T, Liu DC, Zaborina O, Alverdy JC. Red death in Caenorhabditis elegans caused by *Pseudomonas aeruginosa* PAO1. Proc Natl Acad Sci U S A. 2009; 106(15):6327-32.
65. Yang L, Rau MH, Yang L, Høiby N, Molin S, Jelsbak L. Bacterial adaptation during chronic infection revealed by independent component analysis of transcriptomic data. BMC Microbiol. 2011; 11:184.
66. Yang L, Jelsbak L, Marvig RL, Damkiær S, Workman CT, Rau MH, Hansen SK, Folkesson A, Johansen HK, Ciofu O, Høiby N, Sommer MO, Molin S. Evolutionary dynamics of bacteria in a human host environment. Proc Natl Acad Sci U S A. 2011;108(18):7481-6.
67. Rau MH, Hansen SK, Johansen HK, Thomsen LE, Workman CT, Nielsen KF, Jelsbak L, Høiby N, Yang L, Molin S. Early adaptive developments of Pseudomonas aeruginosa after the transition from life in the environment to persistent colonization in the airways of human cystic fibrosis hosts. Environ Microbiol. 2010; 12(6):1643-58.
68. Beaudoin T, Zhang L, Hinz AJ, Parr CJ, Mah TF. The biofilm-specific antibiotic resistance gene ndvB is important for expression of ethanol oxidation genes in *Pseudomonas aeruginosa* biofilms. J Bacteriol. 2012; 194(12):3128-36.
69. Balasubramanian D, Schneper L, Merighi M, Smith R, Narasimhan G, Lory S, Mathee K. The regulatory repertoire of *Pseudomonas aeruginosa* AmpC ß-lactamase regulator AmpR includes virulence genes. PLoS One. 2012;7(3):e34067.
70. Sonnleitner E, Valentini M, Wenner N, Haichar FZ, Haas D, Lapouge K. Novel targets of the CbrAB/Crc carbon catabolite control system revealed by transcript abundance in *Pseudomonas aeruginosa*. PLoS One. 2012;7(10):e44637.
71. Harmer C, Alnassafi K, Hu H, Elkins M, Bye P, Rose B, Cordwell S, Triccas JA, Harbour C, Manos J. Modulation of gene expression by *Pseudomonas aeruginosa* during chronic infection in the adult cystic fibrosis lung. Microbiology. 2013 Nov;159(Pt 11):2354-63.
72. Wei Q, Tarighi S, Dötsch A, Häussler S, Müsken M, Wright VJ, Cámara M, Williams P, Haenen S, Boerjan B, Bogaerts A, Vierstraete E, Verleyen P, Schoofs L, Willaert R, De Groote VN,

Michiels J, Vercammen K, Crabbé A, Cornelis P. Phenotypic and genome-wide analysis of an antibiotic-resistant small colony variant (SCV) of *Pseudomonas aeruginosa*. PLoS One. 2011;6(12):e29276.

73. Williamson KS, Richards LA, Perez-Osorio AC, Pitts B, McInnerney K, Stewart PS, Franklin MJ. Heterogeneity in *Pseudomonas aeruginosa* biofilms includes expression of ribosome hibernation factors in the antibiotic-tolerant subpopulation and hypoxia-induced stress response in the metabolically active population. J Bacteriol. 2012; 194(8):2062-73.
74. Lee KM, Go J, Yoon MY, Park Y, Kim SC, Yong DE, Yoon SS. Vitamin B12-mediated restoration of defective anaerobic growth leads to reduced biofilm formation in *Pseudomonas aeruginosa*. Infect Immun. 2012; 80(5):1639-49.
75. Damron FH, Owings JP, Okkotsu Y, Varga JJ, Schurr JR, Goldberg JB, Schurr MJ, Yu HD. Analysis of the *Pseudomonas aeruginosa* regulon controlled by the sensor kinase KinB and sigma factor RpoN. J Bacteriol. 2012; 194(6):1317-30.
76. Pan J, Bahar AA, Syed H, Ren D. Reverting antibiotic tolerance of *Pseudomonas aeruginosa* PAO1 persister cells by (Z)-4-bromo-5-(bromomethylene)-3-methylfuran-2(5H)-one. PLoS One. 2012;7(9):e45778
77. Lundgren BR, Thornton W, Dornan MH, Villegas-Peñaranda LR, Boddy CN, Nomura CT. Gene PA2449 is essential for glycine metabolism and pyocyanin biosynthesis in *Pseudomonas aeruginosa* PAO1. J Bacteriol. 2013; 195(9):2087-100.
78. Jackson AA, Gross MJ, Daniels EF, Hampton TH, Hammond JH, Vallet-Gely I, Dove SL, Stanton BA, Hogan DA. Anr and its activation by PlcH activity in *Pseudomonas aeruginosa* host colonization and virulence. J Bacteriol. 2013; 195(13):3093-104.
79. Wang J, Dong Y, Zhou T, Liu X, Deng Y, Wang C, Lee J, Zhang LH. *Pseudomonas aeruginosa* cytotoxicity is attenuated at high cell density and associated with the accumulation of phenylacetic acid. PLoS One. 2013;8(3):e60187.
80. Grosso-Becerra MV, Croda-García G, Merino E, Servín-González L, Mojica-Espinosa R, Soberón-Chávez G. Regulation of *Pseudomonas aeruginosa* virulence factors by two novel RNA thermometers. Proc Natl Acad Sci U S A. 2014; 111(43):15562-7.
81. He W, Li G, Yang CK, Lu CD. Functional characterization of the dguRABC locus for D-Glu and d-Gln utilization in *Pseudomonas aeruginosa* PAO1. Microbiology. 2014; 160(Pt 10):2331-40.
82. Lee JH, Kim YG, Cho MH, Lee J. ZnO nanoparticles inhibit *Pseudomonas aeruginosa* biofilm formation and virulence factor production. Microbiol Res. 2014; 169(12):888-96.
83. Bartosik AA, Glabski K, Jecz P, Mikulska S, Fogtman A, Koblowska M, Jagura-Burdzy G. Transcriptional profiling of ParA and ParB mutants in actively dividing cells of an opportunistic human pathogen *Pseudomonas aeruginosa*. PLoS One. 2014; 9(1):e87276.
84. Damron FH, Barbier M, McKenney ES, Schurr MJ, Goldberg JB. Genes required for and effects of alginate overproduction induced by growth of *Pseudomonas aeruginosa* on Pseudomonas isolation agar supplemented with ammonium metavanadate. J Bacteriol. 2013; 195(18):4020-36.
85. Frangipani E, Pérez-Martínez I, Williams HD, Cherbuin G, Haas D. A novel cyanide-inducible gene cluster helps protect *Pseudomonas aeruginosa* from cyanide. Environ Microbiol Rep. 2014; 6(1):28-34.
86. LaBauve AE, Wargo MJ. Detection of host-derived sphingosine by *Pseudomonas aeruginosa* is important for survival in the murine lung. PLoS Pathog. 2014; 10(1):e1003889.

87. Hampel KJ, LaBauve AE, Meadows JA, Fitzsimmons LF, Nock AM, Wargo MJ. Characterization of the GbdR regulon in *Pseudomonas aeruginosa*. J Bacteriol. 2014; 196(1):7-15.
88. Gicquel G, Bouffartigues E, Bains M, Oxaran V, Rosay T, Lesouhaitier O, Connil N, Bazire A, Maillot O, Bénard M, Cornelis P, Hancock RE, Dufour A, Feuilloley MG, Orange N, Déziel E, Chevalier S. The extra-cytoplasmic function sigma factor sigX modulates biofilm and virulence-related properties in *Pseudomonas aeruginosa*. PLoS One. 2013 Nov 18;8(11):e80407.
89. He FQ, Wang W, Zheng P, Sudhakar P, Sun J, Zeng AP. Essential O2-responsive genes of *Pseudomonas aeruginosa* and their network revealed by integrating dynamic data from inverted conditions. Integr Biol (Camb). 2014; 6(2):215-23.
90. Lundgren BR, Villegas-Peñaranda LR, Harris JR, Mottern AM, Dunn DM, Boddy CN, Nomura CT. Genetic analysis of the assimilation of C5-dicarboxylic acids in *Pseudomonas aeruginosa* PAO1. J Bacteriol. 2014; 196(14):2543-51.
91. García-Contreras R, Nuñez-López L, Jasso-Chávez R, Kwan BW, Belmont JA, Rangel-Vega A, Maeda T, Wood TK. Quorum sensing enhancement of the stress response promotes resistance to quorum quenching and prevents social cheating. ISME J. 2015; 9(1):115-25.
92. Zamorano L, Moyà B, Juan C, Mulet X, Blázquez J, Oliver A. The *Pseudomonas aeruginosa* CreBC two-component system plays a major role in the response to β-lactams, fitness, biofilm growth, and global regulation. Antimicrob Agents Chemother. 2014; 58(9):5084-95.
93. Kuroki M, Igarashi Y, Ishii M, Arai H. Fine-tuned regulation of the dissimilatory nitrite reductase gene by oxygen and nitric oxide in *Pseudomonas aeruginosa*. Environ Microbiol Rep. 2014; 6(6):792-801.
94. Vercammen K, Wei Q, Charlier D, Dötsch A, Haüssler S, Schulz S, Salvi F, Gadda G, Spain J, Rybtke ML, Tolker-Nielsen T, Dingemans J, Ye L, Cornelis P. *Pseudomonas aeruginosa* LysR PA4203 regulator NmoR acts as a repressor of the PA4202 nmoA gene, encoding a nitronate monooxygenase J Bacteriol. 2015; 197(6):1026-39.
95. Kim M, Christley S, Khodarev NN, Fleming I, Huang Y, Chang E, Zaborina O, Alverdy JC. *Pseudomonas aeruginosa* wound infection involves activation of its iron acquisition system in response to fascial contact. J Trauma Acute Care Surg. 2015; 78(4):823-9.
96. Stewart PS, Franklin MJ, Williamson KS, Folsom JP, Boegli L, James GA. Contribution of stress responses to antibiotic tolerance in *Pseudomonas aeruginosa* biofilms. Antimicrob Agents Chemother. 2015; 59(7):3838-47.
97. James GA, Ge Zhao A, Usui M, Underwood RA, Nguyen H, Beyenal H, deLancey Pulcini E, Agostinho Hunt A, Bernstein HC, Fleckman P, Olerud J, Williamson KS, Franklin MJ, Stewart PS. Microsensor and transcriptomic signatures of oxygen depletion in biofilms associated with chronic wounds. Wound Repair Regen. 2016; 24(2):373-83.
98. http://msystems.asm.org/content/1/1/e00025-15
99. Xu B, Wozniak DJ. Development of a Novel Method for Analyzing *Pseudomonas aeruginosa* Twitching Motility and Its Application to Define the AmrZ Regulon. PLoS One. 2015; 10(8):e0136426.
100. Willsey GG, Wargo MJ. Sarcosine catabolism in *Pseudomonas aeruginosa* Is transcriptionally regulated by SouR. J Bacteriol. 2015; 198(2):301-10.
101. Guragain M, King MM, Williamson KS, Pérez-Osorio AC, Akiyama T, Khanam S, Patrauchan MA, Franklin MJ. The *Pseudomonas aeruginosa* PAO1 Two-Component Regulator CarSR

Regulates Calcium Homeostasis and Calcium-Induced Virulence Factor Production through Its Regulatory Targets CarO and CarP. J Bacteriol. 2016; 198(6):951-63.

102. McGuffie BA, Vallet-Gely I, Dove SL. σ Factor and Anti-σ Factor That Control Swarming Motility and Biofilm Formation in *Pseudomonas aeruginosa*. J Bacteriol. 2015 Nov 30;198(5):755-65.

103. Reen FJ, Flynn S, Woods DF, Dunphy N, Chróinín MN, Mullane D, Stick S, Adams C, O'Gara F. Bile signalling promotes chronic respiratory infections and antibiotic tolerance. Sci Rep. 2016; 6:29768.

104. Wassermann T, Meinike Jørgensen K, Ivanyshyn K, Bjarnsholt T, Khademi SM, Jelsbak L, Høiby N, Ciofu O. The phenotypic evolution of *Pseudomonas aeruginosa* populations changes in the presence of subinhibitory concentrations of ciprofloxacin. Microbiology. 2016; 162(5):865-75.
